# ERCC1 abundance is an indicator of DNA repair-apoptosis decision upon DNA damage

**DOI:** 10.1038/s41420-024-01817-7

**Published:** 2024-01-25

**Authors:** Sule Erdemir Sayan, Rahul Sreekumar, Rahul Bhome, Alex Mirnezami, Tamer Yagci, A. Emre Sayan

**Affiliations:** 1 Department of Molecular Biology and Genetics, Gebze Technical University, Kocaeli, 41400 Turkey; 2 Cancer Sciences Unit, University of Southampton, Southampton General Hospital, Somers Cancer Research Building, Southampton, SO16 6YD UK

**Keywords:** Nucleotide excision repair, Cancer therapeutic resistance, Apoptosis

## Abstract

DNA repair is essential for successful propagation of genetic material and fidelity of transcription. Nucleotide excision repair (NER) is one of the earliest DNA repair mechanisms, functionally conserved from bacteria to human. The fact that number of NER genes vary significantly between prokaryotes and metazoans gives the insight that NER proteins have evolved to acquire additional functions to combat challenges associated with a diploid genome, including being involved in the decision between DNA repair and apoptosis. However, no direct association between apoptosis and NER proteins has been shown to date. In this study, we induced apoptosis with a variety of agents, including oxaliplatin, doxorubicin and TRAIL, and observed changes in the abundance and molecular weight of NER complex proteins. Our results showed that XPA, XPC and ERCC1 protein levels change during DNA damage-induced apoptosis. Among these, ERCC1 decrease was observed as a pre-mitochondria depolarisation event which marks the “point of no return” in apoptosis signalling. ERCC1 decrease was due to proteasomal degradation upon lethal doses of oxaliplatin exposure. When ERCC1 protein was stabilised using proteasome inhibitors, the pro-apoptotic activity of oxaliplatin was attenuated. These results explain why clinical trials using proteasome inhibitors and platinum derivatives showed limited efficacy in carcinoma treatment and also the importance of how deep understanding of DNA repair mechanisms can improve cancer therapy.

## Introduction

DNA repair (DR) is a fundamental process for all living organisms, governed by distinct but sometimes overlapping pathways, depending on specific insults [1]. Some of these pathways are well-conserved from bacteria to humans. Some others have evolved for the needs of multicellular organisms, i.e. to detect the extent of damage and allow repair or initiate apoptosis [1, 2]. Therefore, DR-apoptosis link is a critical feature of metazoan biology that is necessary to eliminate creating genetically defective progeny.

According to evolutionary conservation, DR pathways can be classified into three main groups [1]. The first group comprises those, present in both prokaryotes and eukaryotes such as direct repair, base excision repair and mismatch repair. In this group, the biochemical processes and proteins involved are highly conserved. The pathways in the second group are eukaryote specific, mostly evolved to combat single-double-strand breaks in DNA such as homologous recombination (HR) and non-homologous end joining (NHEJ). Proteins in HR and NHEJ can also act as sensors of the DNA damage quantity and initiate apoptosis if necessary [2, 3]. This is not surprising as apoptosis has evolved as a eukaryotic feature after mitochondria became a functional organelle [4]. As expected, eukaryote specific post-translational modifications such as caspase processing, ubiquitination and proteasomal degradation, in addition to phosphorylation, are commonly observed in the proteins of the second group (HR, NHEJ), which aligns with the tightly controlled and rapid nature of their job (cell cycle arrest-DNA repair-apoptosis decision) [3, 5]. The third group contains nucleotide excision repair (NER) which evolved to combat DNA damage resulting in distortions of the helical structure of DNA such as UV-induced or chemically formed (e.g. upon cisplatin treatment) base crosslinks [6]. Although the steps and processes of eukaryotic and prokaryotic NER are similar, prokaryotes have only three, whereas eukaryotic NER pathway contains at least nine proteins [7].

These observations suggest eukaryotic NER pathways evolved in parallel with challenges observed in maintaining a complex, multiploid (e.g. diploid) and compartmentalised (chromosomally arranged) genome. However, unlike eukaryote specific DR pathways such as NHEJ, no specific association between a NER component protein and apoptosis has been shown to date. In this article, we will systematically analyse the protein abundance and molecular weight changes (indicative of phosphorylation) of NER complex proteins during the process of apoptosis.

## Results

**Fig. 1:**
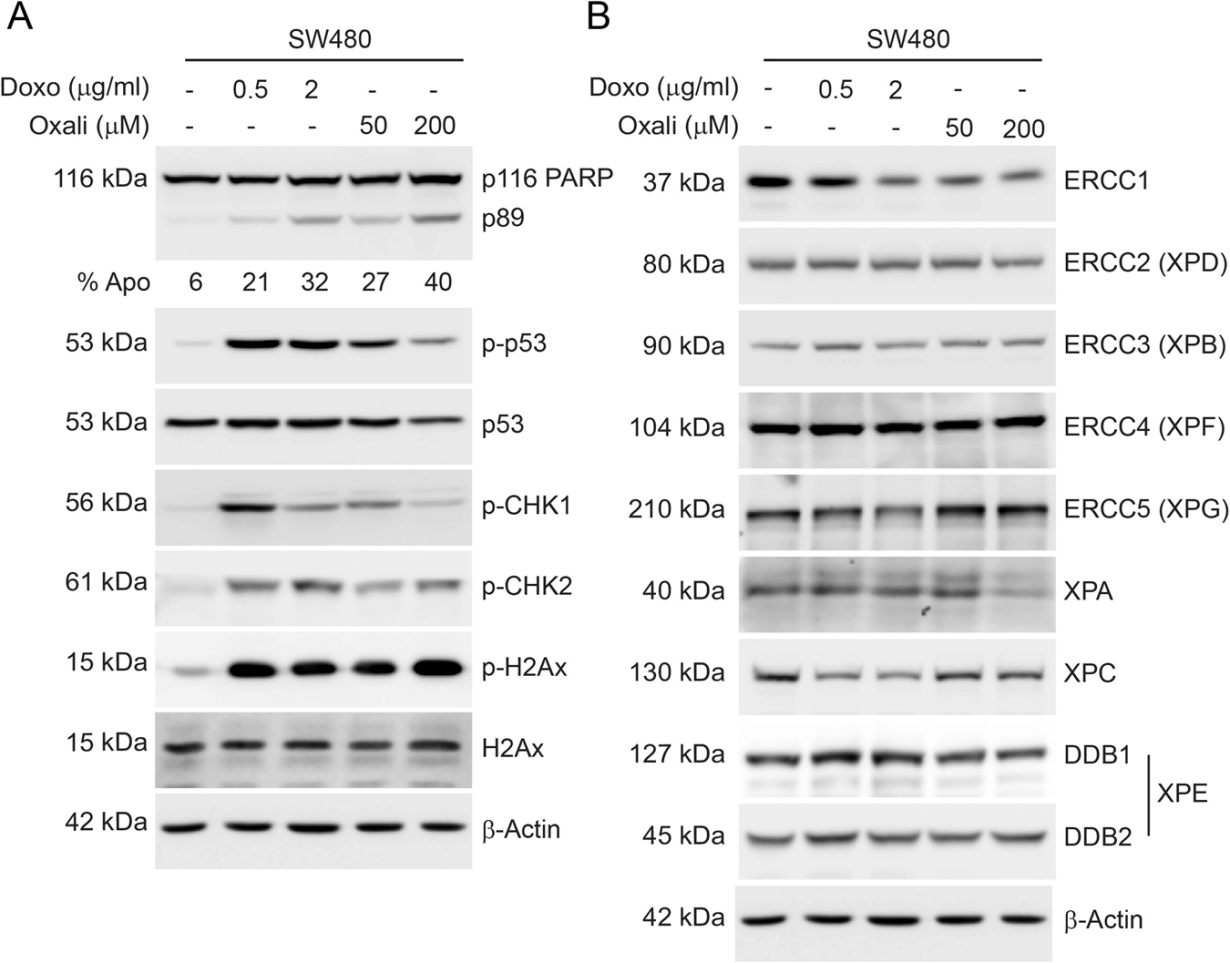
Analysis of NER complementation group proteins during DNA damage induced apoptosis. **A** SW480 cells were treated with doxorubicin and oxaliplatin at indicated amounts for 16 h. Proteins extracted from these cells were subjected to western blotting. Percent Apo at the bottom of PARP western blot indicates sub-G1 (DNA fragmentation analysis) of the same samples. **B** The same proteins from panel A were subjected to western blot analysis for identification of changes in size (molecular weight) and abundance of NER component proteins. The proteins were presented in the order of Excision Repair Cross Complementation (ERCC) identification and naming. Please note ERCC1, XPC and XPA proteins decreased in apoptotic (>%25), doxorubicin treated (irrespective of concentration) and highly apoptotic (>40%) conditions, respectively. As the same cellular lysates were probed in (**A**) and (**B**), the actin blots are the same.

Platinum derivatives such as oxaliplatin are commonly used as chemotherapeutic agents [8]. The damage induced by these drugs is primarily repaired by NER [8]. Doxorubicin is another chemotherapeutic agent, inducing replication fork stalls, which leads to single- or double-strand DNA breaks [9]. Such DNA damage is primarily repaired by HR or NHEJ. There is clinical and biological evidence that both drugs selectively kill rapidly proliferating cancer cells [10]. We initially intended to induce apoptosis in the colorectal cancer cell line SW480 with oxaliplatin or doxorubicin and analyse the abundance of NER component proteins. Crucially, we used 2X Laemmli buffer and sonication to lyse cells and be sure that all proteins were extracted uniformly, whether they are in nucleus, cytoplasm, bound to chromatin or present in another subcellular location. Our results suggest that SW480 cells underwent apoptosis in a concentration dependent manner with these agents as evidenced by cleaved (p89) PARP (Fig. 1A). Both drugs induced phosphorylation of p53, CHK1, CHK2 and the DNA damage marker histone H2Ax (Fig. 1A). Recognition of DNA damage triggers cell cycle arrest and/or apoptosis programmes, which are governed by CHK1 and CHK2, respectively [2]. With both drug treatments a lower concentration induced a higher phosphorylation of CHK1, suggesting activation of cell cycle arrest. On the other hand, higher drug concentration triggered a more pronounced phosphorylation of CHK2, indicating activation of apoptosis. TP53 gene is mutated and presently accumulated in SW480 cells [11], therefore p53 accumulation was not very evident upon DNA damage, however its abundance decreased in line with the extent of apoptosis as it is a caspase target similar to PARP [12]. Phosphorylation of p53 was only detected in cells receiving DNA damage. These results showed we can induce a dose-dependent apoptosis and observe canonical hallmarks of DNA damage response (DDR)-apoptosis pathways in SW480 cells. Next, we investigated the protein abundance of NER component proteins using the same lysates. We observed a decrease in ERCC1 protein at treatments where apoptosis (DNA fragmentation) was more than 25% (Fig. 1B, Supplementary Fig. 1A). XPA protein decreased only with the highest dose of oxaliplatin and correlates with strong apoptosis (Fig. 1B). XPC protein decreased and its migration is retarded only upon doxorubicin treatment, independent of the magnitude of apoptosis. No change of abundance or apparent molecular weight (indicative of extensive phosphorylation) were observed in other NER complex components, ERCC2 (XPD), ERCC3 (XPB), ERCC4 (XPF), ERCC5 (XPG), DDB1 and DDB2. These results suggest that ERCC1 and XPA proteins are potentially downregulated during apoptosis. XPC protein also decreased and is potentially phosphorylated upon doxorubicin treatment.

**Fig. 2:**
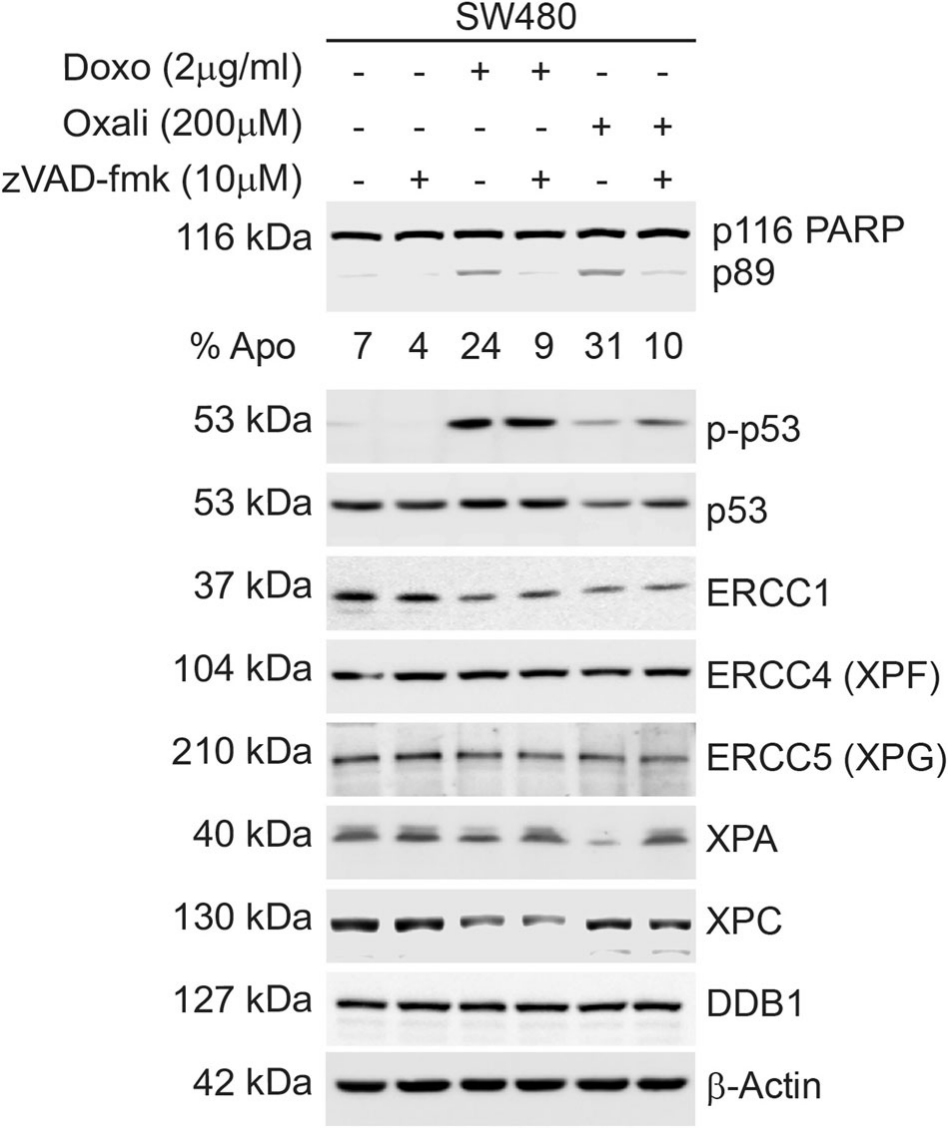
Investigation of caspase activity in the apoptosis induced decrease of NER component proteins. SW480 cells were incubated with high doses of doxorubicin and oxaliplatin for 16 h after a 30 min pre-treatment with the pan-caspase inhibitor zVAD-fmk (10μM). Proteins from floating and attached cells were analysed using western blotting and flow cytometry. Caspase inhibitor effectively inhibited PARP cleavage for both DNA damaging agents as well as DNA fragmentation (sub-G1 DNA formation), as indicated below the PARP blot (% Apo).

A common post translational modification observed during apoptosis is caspase cleavage which results in decreased protein abundance [13]. Majority of caspase substrates lose their function (e.g. PARP) but some are activated to perform a specific function (e.g. DFF45/ICAD) [13]. To investigate the possible involvement of caspases in the decrease of ERCC1, XPC and XPA proteins, we treated SW480 cells with high concentrations of oxaliplatin or doxorubicin and pan-caspase inhibitor zVAD-fmk. Our results suggest zVAD-fmk effectively inhibited caspase cleavage of PARP, as indicated by the absence of p89 (Fig. 2) and DNA fragmentation (Supplementary Fig. 1B). Total- and phospho-p53 protein abundance also recovered with zVAD-fmk in oxaliplatin treated sample. XPA protein abundance returned to untreated levels with zVAD-fmk suggesting it is potentially a caspase target (Fig. 2). ERCC1 protein remained low with both oxaliplatin and doxorubicin, with or without zVAD-fmk treatment, suggesting ERCC1 is not cleaved by caspases (Fig. 2). The decrease observed in XPC in doxorubicin treatment was also not reversed with caspase inhibition suggesting it is a doxorubicin treatment related post-translational event leading to decreased protein levels (Fig. 2). Other NER components (ERCC4, XPG and DDB1) analysed as controls in this assay displayed no change. These results confirmed ERCC1, XPA and XPC as potential proteins modulated during apoptosis. The decrease in XPA most likely yields a loss of function as it is a caspase target. Changes observed in XPC protein abundance and size shift are specific to doxorubicin. The decrease of ERCC1 is consistent in all samples where apoptosis is triggered but not a result of caspase activation. Therefore, ERCC1 is the prime target to be further investigated in DDR-apoptosis link, especially during oxaliplatin-induced apoptosis.

**Fig. 3:**
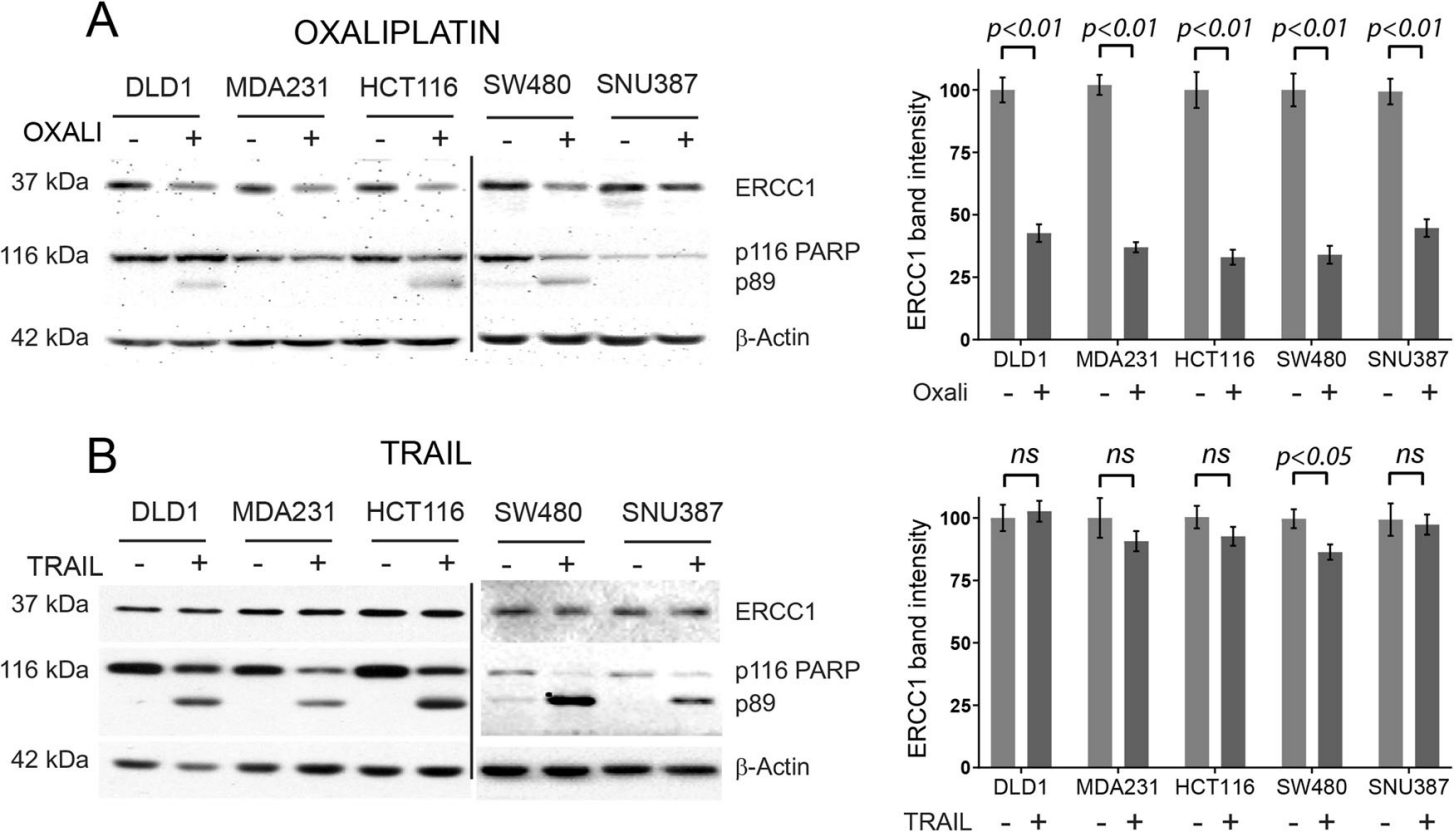
ERCC1 decrease is specific to DNA damage induced apoptosis. A panel of 5 commonly used carcinoma cell lines were treated with oxaliplatin for 16 h (**A**) and death-receptor ligand TRAIL for 6 h (**B**). Apoptosis was assessed by PARP cleavage. ERCC1 decrease was observed exclusively in oxaliplatin (**A**), but not TRAIL (**B**), treated samples. At highly apoptosis resistant cell lines such as MDA231 and SNU387, the decrease in ERCC1 was evident even before PARP cleavage was observed. ERCC1 protein abundance was quantified using ImageJ and normalised against β-actin band, as presented in graph form on the left side of each panel. Statistical significance was assessed using paired student *t*-test and indicated in graphs for both panels. A *p* value less than 0.05 is considered significant, ns non-significant. The graph represents standard error of mean for 3 independent experiments.

Next, we investigated if our observations were specific to DNA damage-induced apoptosis. To generalise our findings, we treated five commonly used cancer cell lines of colorectal (DLD1, HCT116 and SW480), breast (MDA231) and hepatocellular carcinoma (SNU387) origin with oxaliplatin to activate caspase-9, or death-receptor ligand TRAIL to activate caspase-8,-initiated apoptosis. Our results confirmed our previous observations that ERCC1 abundance decreases with high concentration of oxaliplatin (Fig. 3A). Importantly, highly oxaliplatin resistant cell lines such as MDA231 and SNU387 [14] had decreased ERCC1 levels with minimal PARP cleavage, suggesting ERCC1 decrease may be an event, happening prior to caspase activation, similar to CHK2 phosphorylation or p53 accumulation during apoptosis cascade. Death-receptor stimulation by TRAIL did not yield any decrease in ERCC1 protein abundance despite inducing strong apoptosis, as evidenced by extensive PARP cleavage (Fig. 3B). These results suggest ERCC1 decrease is a DNA damage-induced apoptosis-specific event.

**Fig. 4:**
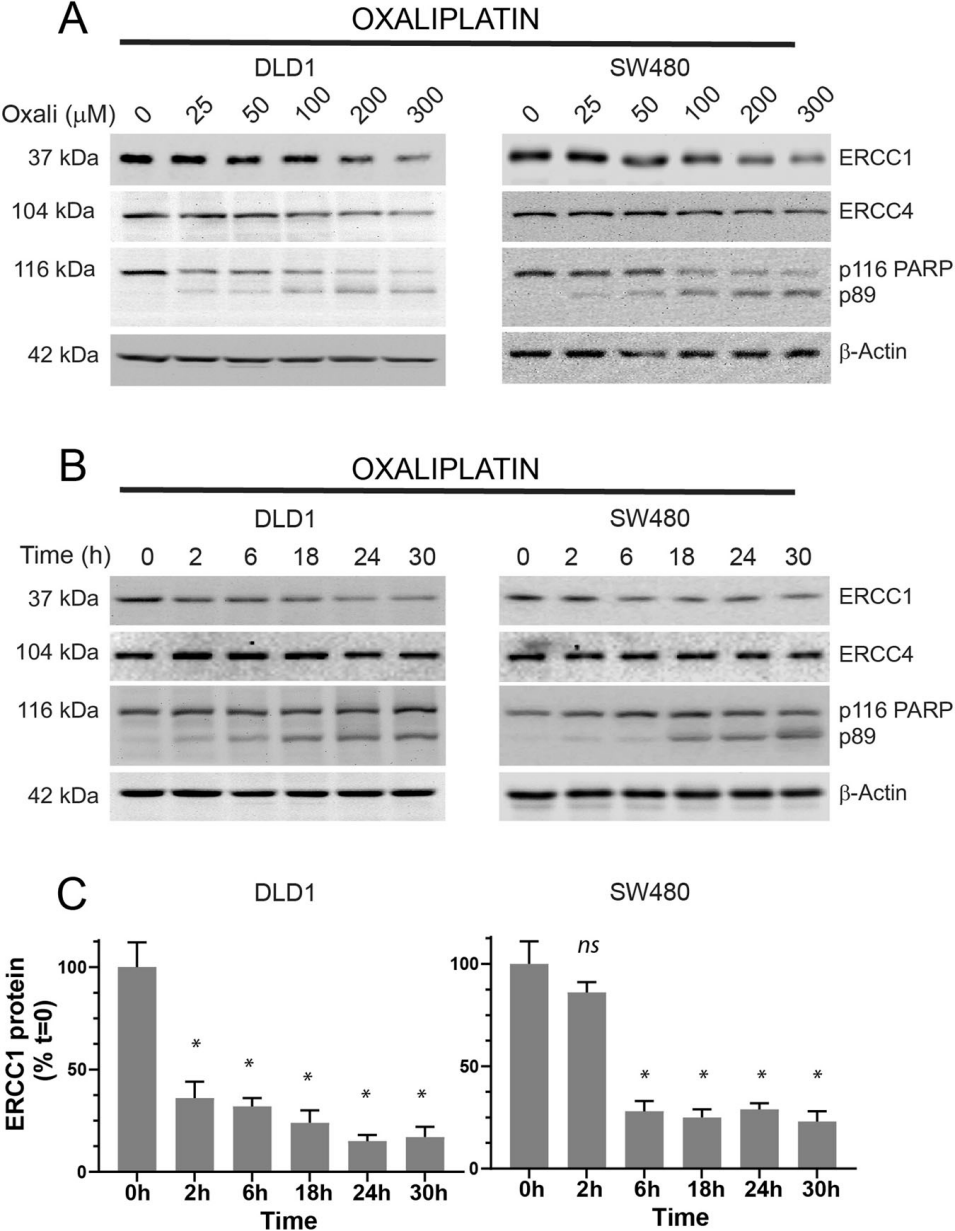
Concentration and time dependent decrease in ERCC1 protein. **A** DLD1 and SW480 cell lines were treated with increasing (25–300 μM) concentrations of oxaliplatin for 16 h. PARP cleavage was used to identify the extent of apoptosis and ERCC4 protein was assessed as a NER component, XPF complex protein and control. **B** DLD1 and SW480 cell lines were treated with 200 μM oxaliplatin for 2–30 h. The decrease in ERCC1 was observed at the 2 h in DLD1 and at 6 h in SW480 cells, before or at the earliest detection of PARP cleavage. **C** Equal quantity of proteins from samples of the time curve experiment (panel **B**) were subjected to ERCC1-enzyme linked immune-absorbance assay (ELISA). The quantity of ERCC1 protein at time = 0 is considered 100%. The measured ERCC1 protein concentration at *t* = 0 for DLD1 cells is 264 pg/ml and for SW480 cells is 811 pg/ml. Paired Student *t-*test is used to identify statistically significant changes as compared to t = 0. A *p* value less than 0.05 is considered significant and indicated with a “*”. ns non-significant. The graphs represents standard error of mean for 3 independent experiments.

As we confirmed the role of oxaliplatin in reducing ERCC1 protein levels, we wanted to address the correlation between the concentration of oxaliplatin, exposure time and magnitude of apoptosis with ERCC1 protein abundance. To achieve our aim, we treated two cell lines (DLD1 and SW480) with increasing concentrations of oxaliplatin (25–300 μM) for 16 h and assayed for ERCC1, ERCC4 and PARP. We observed that ERCC1, but not ERCC4, is decreasing where apoptosis became evident, at a range of 100–200 μM (Fig. 4A). As apoptosis is a progressive event, we additionally performed a time curve with the same cells, ranging from 2–30 h with a high concentration (200 μM) of oxaliplatin. This time, the decrease in ERCC1 became evident before significant PARP cleavage (e.g. 6 h, in SW480 cells) was detectable (Fig. 4B). Quantitative detection of ERCC1 protein in these conditions using ELISA technique confirmed a significant decrease as early as 2 h in DLD1 and 6 h in SW480 cells (Fig. 4C). These results suggest ERCC1 may be decreasing before mitochondria depolarisation (caspase activation) as also observed in Fig. 3A (MDA231 and SNU387 cells) suggestive of a commitment display to DNA damage-induced apoptosis.

**Fig. 5:**
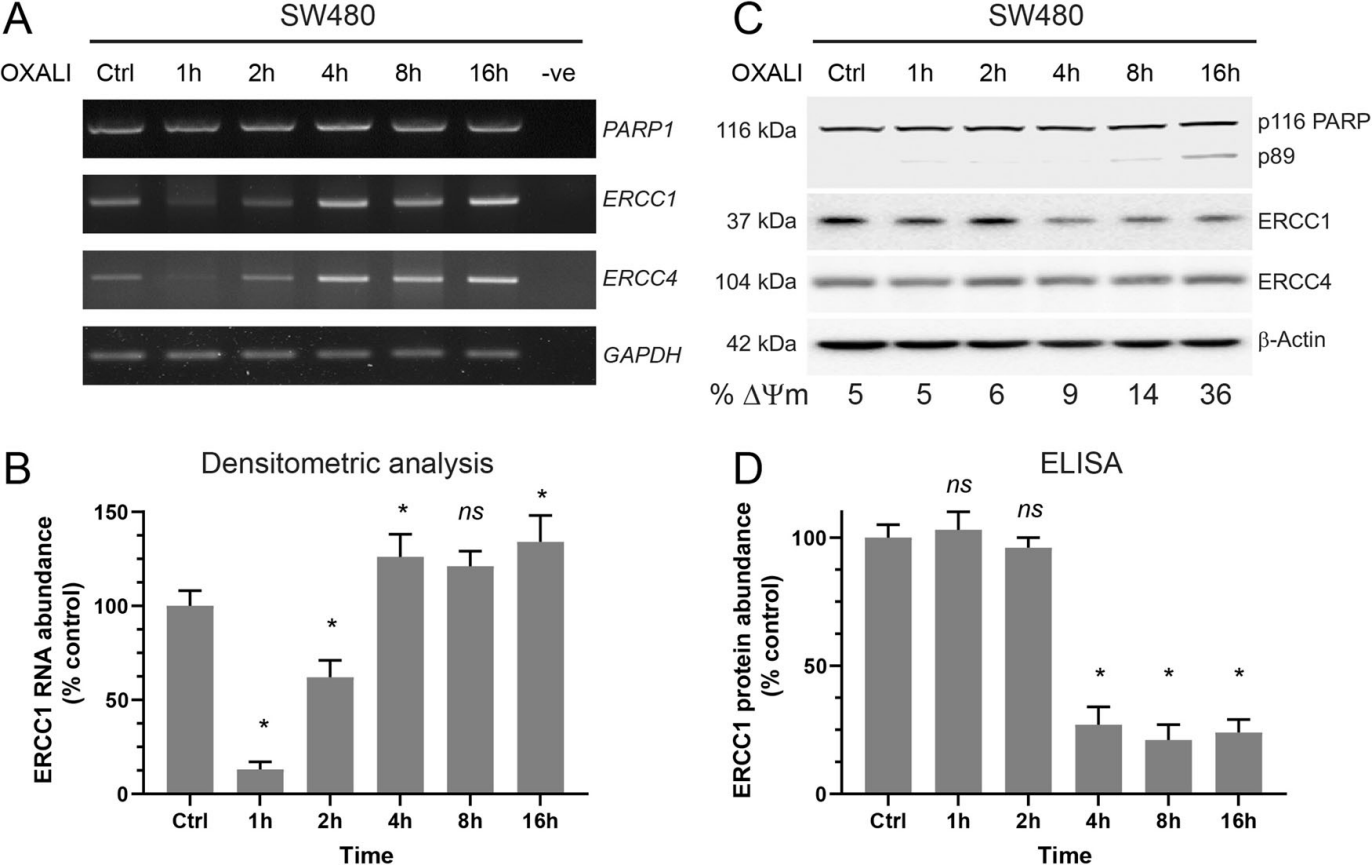
Kinetics of ERCC1 gene and protein expression upon oxaliplatin treatment. SW480 cells were treated with 100 μM oxaliplatin for the indicated time periods, cells (floating and attached) were collected and subjected to RNA extraction (50% of total), protein extraction (40% of the total) and flow cytometry (10% of total). **A** Semi-quantitative RT-PCR analysis of *PARP1*, *ERCC1* and *ERCC4* genes suggest *PARP1* levels do not change as a result of oxaliplatin treatment, whereas *ERCC1* and *ERCC4* genes show a sudden dip till 2 h and recovery from 4 h onwards. **B** ERCC1 bands from panel **A**, obtained from 3 independent experiments, were quantified using ImageJ and presented as a graph. The intensity of ERCC1 band at time 0 is considered 100%. Paired Student *t-test* is used to identify statistically significant changes as compared to *t* = 0. A *p* value less than 0.05 is considered significant and indicated with a “*”. ns non-significant. **C** Western blot analysis of the above mentioned samples show a decrease of ERCC1 protein at 4 h and onwards. PARP protein is cleaved starting from 8 h, coinciding with mitochondria depolarisation (ΔΨm), shown at the bottom of panel **C**. ERCC4 protein is used as control. **D** Equal quantity of proteins from samples presented in panel **C** were subjected to ERCC1-enzyme linked immune-absorbance assay (ELISA). The quantity of ERCC1 protein at time 0 is considered 100% as measured 833 pg/ml. Paired Student *t-*test is used to identify statistically significant changes as compared to *t* = 0. A *p* value less than 0.05 is considered significant and indicated with a “*”. ns non-significant. The graph represents standard error of mean for 3 independent experiments.

Changes in protein abundance can be a reflection of transcriptional, translational or post-translational regulations. To precisely time the decrease of ERCC1 protein during oxaliplatin-induced apoptosis and to see whether ERCC1 transcription or translation is altered in that process, we incubated SW480 cells with 200μM oxaliplatin and collected cells at early (1, 2, 4 h) and late (8 and 16 h) time points. Analysis of *ERCC1* gene expression showed a sudden dip at 1 h and recovery from 4 h onwards (Fig. 5A, B). *ERCC4* gene showed a similar trend. Other genes analysed in this assay, involved in DR-apoptosis (*PARP1*) or *GAPDH* (equal loading control) did not change during the course of the experiment. Importantly, when proteins from the same time points were analysed, the downregulation of ERCC1 protein was evident at 4 h whereas ERCC4 displayed no change despite showing a similar trend of RNA expression (Fig. 5C). PARP cleavage was initiated at 8 h and became evident at 16 h, which coincided both temporally and magnitude-wise with mitochondria depolarisation (as indicated at the bottom of Fig. 5C). Further, we performed ELISA to assess ERCC1 protein levels in these conditions, in a quantitative manner. Our results support previous observations and point to a significant and sustained ERCC1 decrease, starting 4 h after oxaliplatin treatment (Fig. 5D). These results confirmed ERCC1 protein abundance is controlled post-translationally and its downregulation is an early event in DNA damage-induced apoptosis, happening prior to mitochondria depolarisation.

**Fig. 6:**
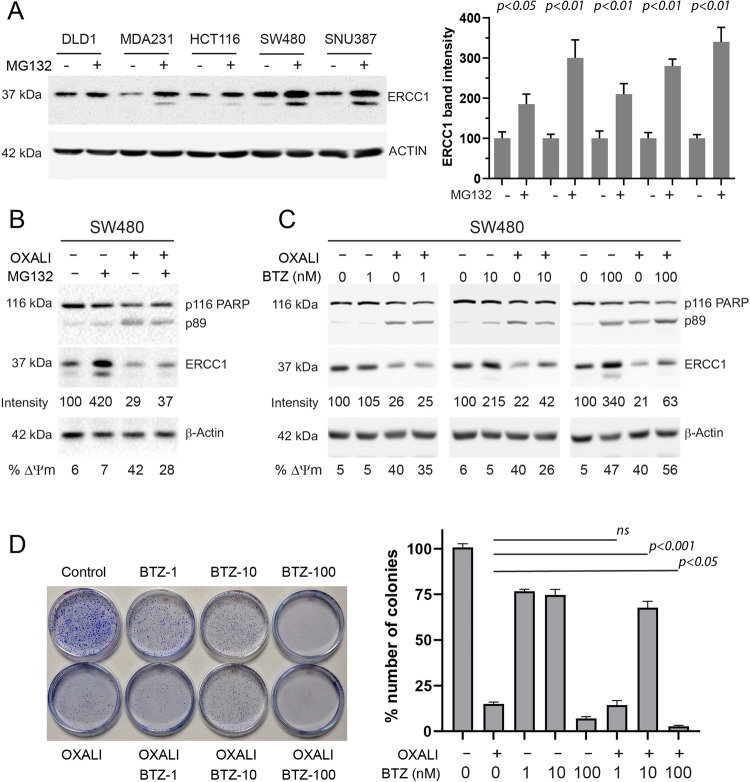
Modulation of ERCC1 protein abundance through proteasomal degradation and its cellular and clinical relevance. **A** Five carcinoma cell lines were treated with the proteasome inhibitor MG132 (5 μM) and ERCC1 protein abundance analysed 6 h later by western blotting (left panel). ERCC1 bands from 4 different experiments were quantified using ImageJ and presented as a graph (right panel). Paired student *t*-test was used to identify statistical significance and indicated on the top of the graph for each pair of samples. A *p*-value less than 0.05 is considered significant. **B** SW480 cells were pre-treated with 0.5μM MG132 for 4 h and then incubated with 200μM oxaliplatin for 16 h. Oxaliplatin-induced cell death was evident with the appearance of cleaved PARP band and as observed by mitochondria depolarisation (ΔΨm, as indicated at the bottom of the figure). ERCC1 band intensity was measured using ImageJ and presented under ERCC1 blot. **C** SW480 cells were pre-treated with different concentrations of Bortezomib (BTZ) for 6 h and further incubated with oxaliplatin (200μM) for 16 h. Proteins from these samples were probed for ERCC1 abundance and PARP cleavage (as indicator of apoptosis). BTZ facilitated ERCC1 accumulation and also decreased apoptosis response in non-toxic (10 μM) concentration (middle panels). ERCC1 band intensity was measured using ImageJ and presented under ERCC1 blot. Mitochondria depolarisation (ΔΨm) is indicated at the bottom of the figure. **D** Long term effects of oxaliplatin and BTZ in cell viability as assessed by colony formation assay. Cells seeded at low density (2000 cells/6 cm dish) were treated with 1, 10 or 100 nM BTZ for 6 h and then 1μM oxaliplatin is added onto cells. The colonies formed after 14 days were stained, visualised and counted. The dishes were presented as left and quantification of colonies were presented as right panels. The dose of BTZ that is relatively non-toxic to cells (10 nM) and stabilising ERCC1 significantly (10 nM) increases the viability of cells, treated with lethal dose of oxaliplatin. The significance between oxaliplatin alone and BTZ-oxaliplatin co-treatments, as assessed by paired student *t-test*, were presented in the graph. A *p* value less than 0.05 is considered significant. Number of colonies in control plates were taken as 100%. The graph represents standard error of mean for 3 independent experiments.

Ubiquitin-dependent proteasomal degradation is the major cellular pathway controlling protein stability [15]. ERCC1 has been shown to be ubiquitylated and subjected to proteasomal degradation however its E3 ligase, and its ubiquitylation site/s have not been addressed before. Most importantly, despite ERCC1 function correlating with chemotherapy response in carcinoma (e.g. lung, bladder, ovarian cancers), only non-carcinoma (melanoma or sarcoma) cell lines were used in the two previous studies focusing on ERCC1 degradation/ubiquitylation [16, 17]. To investigate the mechanism of ERCC1 decrease and its functional contribution to apoptosis, we incubated our five carcinoma cell line panel with the proteasome inhibitor MG132 and confirmed ERCC1 levels are go up, similar to melanoma and sarcoma (Fig. 6A). Further, we treated SW480 cells with oxaliplatin with or without MG132 pre-incubation. Our results suggest ERCC1 increase hinders oxaliplatin-induced apoptosis as shown by reduced mitochondria depolarisation and PARP cleavage (Fig. 6B). Of note, proteasome inhibitors such as Bortezomib are used as chemotherapeutic agents in haematological malignancies, but never showed efficacy in carcinomas as part of a platinum containing combination therapy [18]. We hypothesised that increased ERCC1 abundance upon proteasome inhibition could be a reason for negative results obtained in clinical trials involving Bortezomib and oxaliplatin. Therefore, we incubated SW480 cells with low, medium and high doses of Bortezomib in combination with oxaliplatin, assessed ERCC1 abundance and biochemical hallmarks of apoptosis. Our results suggest Bortezomib does not induce apoptosis at low concentrations (1 nM) by itself or alter oxaliplatin-induced apoptosis, neither has it caused ERCC1 accumulation (Fig. 6C). ERCC1 increase became evident at a higher concentration of Bortezomib (10 nM), and it induced detectable apoptosis. Importantly, cells treated with Bortezomib and oxaliplatin displayed less apoptosis than oxaliplatin alone suggesting ERCC1 stabilisation blocked the activity of oxaliplatin (Fig. 6C, middle panel). At the highest concentration tested (100 nM), Bortezomib induced significant apoptosis and when combined with oxaliplatin, the magnitude of cell death was even more, despite ERCC1 accumulation (Fig. 6C, right panel). These results suggest Bortezomib and oxaliplatin kill cells using different mechanisms and only synergise in pro-apoptotic activity when used at very high concentrations. Colony formation assay using three different concentrations of Bortezomib, with or without oxaliplatin, confirmed our results (Fig. 6D). To summarise, ERCC1 stabilisation upon proteasome inhibition hinders oxaliplatin-induced apoptosis.

## Discussion

NER is one of the earliest DNA repair pathways, a process conserved from bacteria to metazoans [7]. In humans, mutations in NER genes result Xeroderma Pigmentosum (XP) syndrome, which presents with increased sensitivity to UV and risk of skin cancer [19]. NER has evolved to eliminate helix-altering DNA distortions such as pyrimidine dimers, strand crosslinks and bulky adducts. Despite the actual repair process being similar in all organisms, eukaryotic NER can employ different proteins in the damage recognition process. Global-genomic (GG-NER) is mainly responsible in repairing non-coding regions of DNA and Transcription-coupled (TC-NER) mostly deals with insults at actively coding regions [20]. In GG-NER, XPC identifies DNA helix distortion and binds to the opposite strand, dislocating, and exposing damaged bases/basepairs. The fact that XPC complex is not recognising a specific insult but a distortion and that it binds to the opposite (normal) strand makes GG-NER very versatile. Then, TFIIH-XPB-XPD helicase complex binds the damaged region and unwinds the distorted DNA. This complex stalls at the damaged site where XPA joins the complex to mark the precise helix distorting nucleotide dimer. After that, XPG and ERCC1-XPF nucleases make incisions at 3′- and 5′-sites, respectively, which results in the removal of a 20–30 base containing single strand oligo-nucleotide spanning the damaged base/s. The gap is filled by de novo DNA synthesis and the nick at the 3′-end is sealed by DNA ligase, which completes the GG-NER process. Certain DNA adducts such as UV-induced cyclobutane pyrimidine dimers (CPD) do not cause major helix distortions. In such cases, DDB1 and DDB2 proteins (XPE complex) bind to the damaged site and recruit XPC to start NER. TC-NER does not require XPE or XPC to detect DNA adducts. Stalled RNA polymerase-II serves as a platform for the recruitment of TFIIH and NER machinery excises the segment of mutated DNA.

As described above, eukaryotic NER operates through a series of protein-protein interactions in a stoichiometrically well-defined context. For example, binding of one XPG and one XPF (ERCC1-XPF) proteins to the core NER complex is necessary for 3′ and 5′ excision of the damaged oligonucleotide, respectively [6, 20]. However, silencing genes involved in 5′ exonuclease activity of NER complex (ERCC1/XPF) yields a much more pronounced sensitisation to apoptosis when compared to the 3′ exonuclease (XPG gene) upon UV treatment [21]. XPG-mutant patients display Cockayne syndrome symptoms whereas XPF (ERCC4) mutations yield Fanconi anaemia-like features in addition to common XP manifestation [22]. There is also evidence that ERCC1/XPF play a role in eukaryotic DNA maintenance/rearrangement events such VDJ recombination or telomere maintenance similar to eukaryote specific DR pathways [23]. These findings suggest NER complex proteins are also involved in other DR processes which can link them to DR-apoptosis decision.

Among all NER proteins, only XPC and DDB2 have been studied regarding post-translational modifications [19]. XPC phosphorylation at serine-94 by casein kinase 2 (CK2) increases its binding to damaged DNA [24]. Serine-892 of XPC was also shown to be phosphorylated with a positive impact on NER activity but the relevant kinase is under debate [25]. Both ubiquitylation and sumoylation increase the stability of XPC protein in addition to increasing its affinity to damaged DNA, especially to CPD lesions [26, 27]. CRL4 complex (cullin4A and RBX1 core ubiquitin ligase) bound to XPE (DDB1 and DDB2) is necessary for XPC ubiquitylation. DDB1 serves as an adaptor for CRL4 substrate binding and XPC is brought to this complex by DDB2. Importantly, DDB2 is also ubiquitylated by CRL4 complex which results in its degradation, releasing XPC to initiate GG-NER upon UV treatment [28].

In this article, we investigated the changes in protein abundance of all NER complex components during apoptosis. We observed XPC protein decrease upon doxorubicin treatment; it was not dependent on the magnitude of apoptosis and cannot be reversed by caspase inhibition. The damage induced by doxorubicin (ssDNA and dsDNA breaks) is not repaired by NER pathway, therefore the impact or contribution of XPC to this process remains unclear and requires further attention. XPA, on the other hand, decreased when significant (more than 25%) apoptosis was observed and can be restored by the caspase inhibitor zVAD-fmk. This suggests XPA is a caspase target, similar to other DR proteins such as ATM [29]. XPA elimination should stop NER process once the damage is deemed unrepairable. Most importantly, we observed a decrease in ERCC1 abundance upon DNA damage, but not TRAIL-induced, apoptosis. From dose and kinetic experiments, we concluded that ERCC1 reduction precedes mitochondria depolarisation, which is the point of no-return in the process of intrinsic apoptosis [13]. RNA levels of *ERCC1* did not correlate with its protein abundance, suggesting this regulation is at post-translational level. ERCC1 can be stabilised by proteasome inhibition, which limits its degradation upon oxaliplatin treatment and protects cells from apoptosis. Previous reports suggest ERCC1 protein is subjected to ubiquitination however its E3 ligase and positions of lysines modified are still at large [16, 17]. Its deubiquitinylase has been discovered as USP45. Published data suggest that ERCC1 half-life does not increase in USP45 knock out cells, suggesting ubiquitination is controlling its function, rather than abundance [16]. This brings up the possibility that ERCC1 can be degraded by proteasome through a proteasome-dependent but ubiquitylation-independent manner. For example, Fra-1 protein has a c-terminal degron that associates with 19S proteasome, unless phosphorylated [30, 31]. A DNA-damage-induced kinase could be phosphorylating ERCC1 to be targeted for proteasomal degradation. The same kinase can be phosphorylating other proteins to initiate events upstream of mitochondria depolarisation. Therefore, ERCC1 decrease or no-decrease can mark “die or repair” decision, respectively. It is important to identify upstream signalling pathways and molecular mechanisms leading to ERCC1 decrease.

The modulation of ERCC1 in DDR and the possibility that it represents a decision point between cell survival and apoptosis can have critical impact in chemotherapy response in cancer patients. ERCC1 has been put forward and tested extensively to predict platinum response in cancer therapy, with occasionally positive results [32]. Among all NER component genes, the absence of *ERCC1* produces the most chemosensitive phenotype, inducing a 10 fold increase in UV-induced apoptosis [21]. A cellular trans-differentiation programme called Epithelial-Mesenchymal Transition (EMT) was shown to activate *ERCC1* expression with acquired resistance to oxaliplatin-induced apoptosis and clinically detectable consequences in terms of adjuvant chemotherapy response [33]. EMT is critical for the formation of metastatic cells or cancer cells with stem cell properties, which are slowly proliferating, motile, and apoptosis resistant [34–37]. In metastatic or high ERCC1 expressing cancer cells, downregulation of ERCC1 reversed chemoresistance against oxaliplatin or cisplatin [33, 38]. Excellent response to cisplatin in testicular cancer patients was explained by the inherent low ERCC1 expression in these tumours [39]. These findings suggest the modulation of ERCC1 protein abundance can impact chemotherapy response.

Here, we showed that Bortezomib protects cells from oxaliplatin-induced apoptosis at concentrations stabilising ERCC1, therefore acting in an antagonistic manner. Only at higher concentrations, these two chemotherapeutic agents synergised. Despite encouraging results from in vitro studies, clinical trials of Bortezomib in carcinomas in combination with conventional chemotherapy failed [18]. Importantly, our findings can explain what is observed in patients as the antagonistic action of Bortezomib and oxaliplatin can be observed at the middle, but not low or high, doses in several early phase trials [40]. Therefore, the outcome of cancer therapy could be influenced by chemotherapeutic agents controlling ERCC1 protein stability such as Bortezomib. The biology of NER is critically important for identifying new treatment strategies.

## Materials and methods

### Cell lines and reagents

DLD1, HCT116, SW480, MDA231 and SNU387 cells were obtained from ATCC and validated using STR analysis (Eurofins, Luxembourg). All cells were propagated in DMEM with 10%FCS in a humidified incubator with 5% CO_2_. Colony formation assay was performed as described before [14]. Apoptosis inducing agents, proteasome inhibitors, caspase inhibitor and other reagents are listed in supplementary table 1.

### Apoptosis assays

Apoptosis detection was always performed using more than one technique according to published recommendations [13]. Flow cytometry was used to assess apoptosis in a quantitative, and western blotting for qualitative, manner. Briefly both floating and attached cells were collected after exposure to pro-apoptotic agents. Twenty percent of the cells was subjected to flow cytometry for either sub-G1 analysis (using RNAse and PI for experiments involving doxorubicin) or mitochondria depolarisation (using TMRE for experiments involving non-fluorescent apoptosis triggers such as TRAIL or oxaliplatin) [34]. The rest (80%) of the cells were lysed and proteins subjected to western blotting to observe PARP cleavage (as an indicator of effector caspase activity). Cells were incubated with DNA damaging agents for 16 h and TRAIL for 6 h unless indicated otherwise.

### Western blotting

Cell lysates were prepared by solubilising the cell pellet in 2X Laemmli buffer and sonication as described before [34]. Briefly, 20 μg of protein was separated under reducing conditions in SDS-PAGE gels and transferred onto nitrocellulose membranes (Amersham, 10600000), blocked in 5% non-fat dry milk, and then incubated with primary antibodies, as listed in supplementary table 2. Membranes were washed and incubated with HRP-conjugated secondary antibodies (anti-rabbit, P021702-2, anti-mouse, P016102-2, Dako, anti-goat, SC2020, SantaCruz); in 1:3000 dilution. The specific signal was visualised using chemi-luminescent detection kit (Supplementary Table 1). Where necessary, band intensity was quantified using Image J. Values shown are relative to β-actin.

### Enzyme Linked Immuno-absorbance assay (ELISA)

Cell extracts, prepared as in western blotting section, were diluted to 250 μg/ml (from 2–3 mg/ml stock) using dilution buffer as recommended by the supplier of kit (Elabscience, EL-H1853) to be in the linear range of detection. The protocol involves adding samples or standards to the wells (2 wells for each sample, 3 biological duplicates), incubation of 30 min at RT followed by addition of detection antibody mix. Following an incubation of 30 min, the unbound material was removed by 3 washes and detection was achieved using a TMB based colorimetric assay and Victor plate reader (405 nm detection/600 nM background). Recombinant ERCC1 protein was used as a standard in tripling concentrations at 5 concentration intervals (80, 240, 720, 2160 and 6480 pg/ml).

### Gene expression analysis

Total RNA was isolated using Qiazol reagent (Qiagen, 79306); quality and concentrations were determined using NanoDrop 8000 (ThermoFisher). Total RNA (2 μg) was converted into cDNA using RevertAid cDNA synthesis kit (ThermoFisher, K1622). cDNA, was, then, diluted 1/3 and 2μl is used as template. PCR reagents, primers, semi-quantitative cycle number, annealing temperature and amplicon sizes are listed in Supplementary Tables 1, 3.

### Statistical analysis

Where individual images (e.g. western blotting) are displayed, these are representative of at least three independent experiments. Graphics represent the mean ± SEM, unless otherwise stated. The differences in mean-relative values were tested by two-tailed paired t-test. The threshold level of significance was set at 0.05 for all statistical tests.

### Supplementary information

Erdemir et al. supplementary info

Original Data File

## Data Availability

The data used to support the findings of this study are available from the corresponding author upon request.
