# Supplementary material for: ERCC1 abundance is an indicator of DNA repair-apoptosis decision upon DNA damage
Source: Cell Death Discov. 2024 Jan 25;10:47. doi: 10.1038/s41420-024-01817-7 (PMC10810800; doi:10.1038/s41420-024-01817-7)
Supplement: Supplementary file 1 — Erdemir et al. supplementary info [file 41420_2024_1817_MOESM1_ESM.pdf]

ERCC1 abundance is an indicator of DNA repair-apoptosis decision upon DNA damage.

Sule Erdemir Sayan [1], Rahul Sreekumar [2], Rahul Bhome [2], Alex Mirnezami [2], Tamer Yagci [1] and A. Emre Sayan [2, *].

Supplementary information:

Table 1: Reagents used in this study, company names and catalogue numbers.

Reagent	Company	Catalog No.
**Oxaliplatin**	Hospira UK	-
**Doxorubicin**	Sigma	D1515
**z-VAD-FMK**	Merck	627610
**MG-132**	Merck	474787
**Bortezomib**	Merck	5043140001
**TMRE**	Invitrogen	T669
**Immobilon Forte Western HRP substrate**	Merck	WBLUF0100
**MyTaq Red Mix**	Meridian Bioscience	BIO-25043
**TRAIL**	Peprotech	310-04

Table 2: Antibodies used in this study, company names, catalogue numbers, dilutions and molecular weight (MW) of the target protein.

Antibody	Company	Catalog No.	Dilution	MW (kDa)
**PARP**	Cell Signaling	9542S	1:1000	116, 89
**p-p53**	Cell Signaling	9286P	1:1000	53
**p53**	Santa Cruz Biotechnology	SC-6243	1:300	53
**p-CHK1**	Cell Signaling	2348P	1:500	56
**p-CHK2**	Genetex	GTX132204	1:500	61
**p-H2A.X**	Cell Signaling	2577S	1:3000	15
**H2A.X**	R&D Systems	MAB3406	1:300	15
**β-Actin**	BD Biosciences	612656	1:3000	42
**ERCC1**	Cell Signaling	12345S	1:1000	37
**ERCC2(XPD)**	Abclonal	A19241	1:500	80
**ERCC3(XPB)**	Abclonal	A12702	1:500	90
**ERCC4(XPF)**	Cell Signaling	13465S	1:500	104
**ERCC5(XPG)**	Genetex	GTX110609	1:500	210
**XPA**	R&D Systems	AF3416	1:500	40
**XPC**	Abclonal	A8354	1:500	130
**DDB1**	Elabscience	E-AB-12364	1:500	127
**DDB2**	Abclonal	A11615	1:1000	45

Table 3: Primers used in this study, amplicon size, annealing temperature and semi-quantitative cycle number.

Gene	Forward primer (5’-3’)	Reverse primer (5’-3’)	Amplicon size	T_m	Cycle number
PARP1	TTTGGGCAAAACTACCCCTG	TACCCATCAGCAACTTAGCGG	294 bp	60°C	28
ERCC1	CTCCCGGGTGACTGAATGTC	TTCAGAGTCTGGGGAGGAGG	445 bp	60°C	31
ERCC4	CATCCATCCGCTTCTGGGTT	GACATGGAGATGCACTGGCT	588 bp	60°C	31
GAPDH	GGCTGAGAACGGGAAGCTTGTCAT	CAGCCTTCTCCATGGTGGTGAAGA	143 bp	60°C	26

SUPPLEMENTARY FIGURE 1

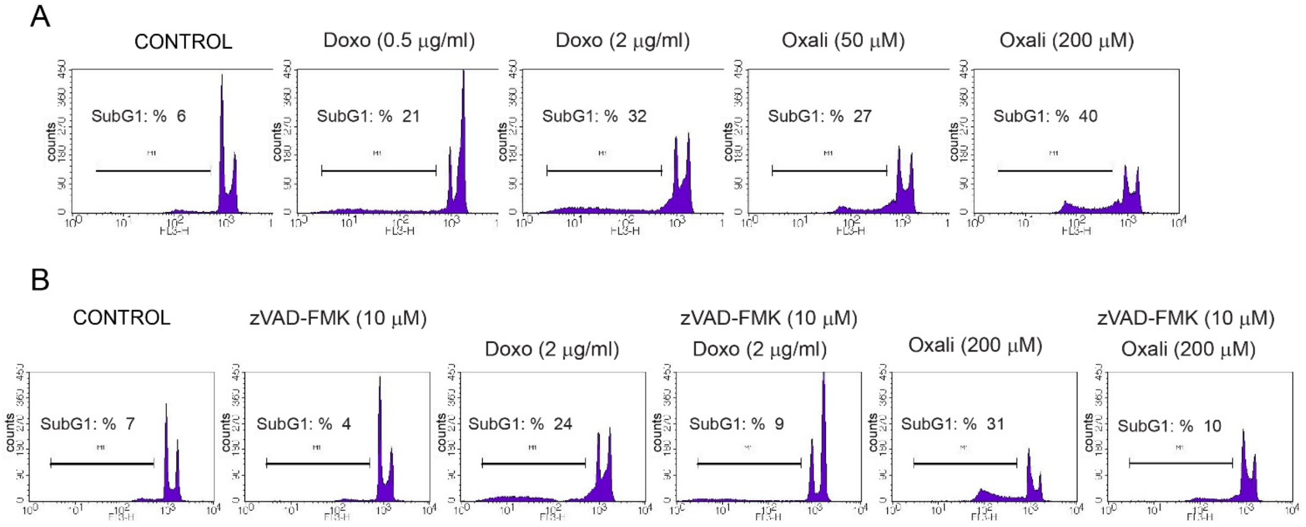

Supplementary Figure 1: Genotoxic drug induced DNA fragmentation analysis **(A)** SW480 cells were treated with Doxorubicin (0.5-2 μg/ml) or oxaliplatin (50-200μM) for 16h and subjected to propidium iodide staining. Presence of DNA with staining intensity less than the G1 peak, as indicated with M1, registers as apoptosis (SubG1 DNA) and indicated in individual panels. **(B)** SW480 cells were pre-treated with caspase inhibitor zVAD-FMK for 30 mins and incubated 16h with Doxorubicin or oxaliplatin. SubG1 analysis of was performed as indicated in previous panel. zVAD-FMK effectively inhibits effector caspase activation and DNA fragmentation.
