# Supplementary material for: ERCC1 abundance is an indicator of DNA repair-apoptosis decision upon DNA damage
Source: Cell Death Discov. 2024 Jan 25;10:47. doi: 10.1038/s41420-024-01817-7 (PMC10810800; doi:10.1038/s41420-024-01817-7)

Figure 1A

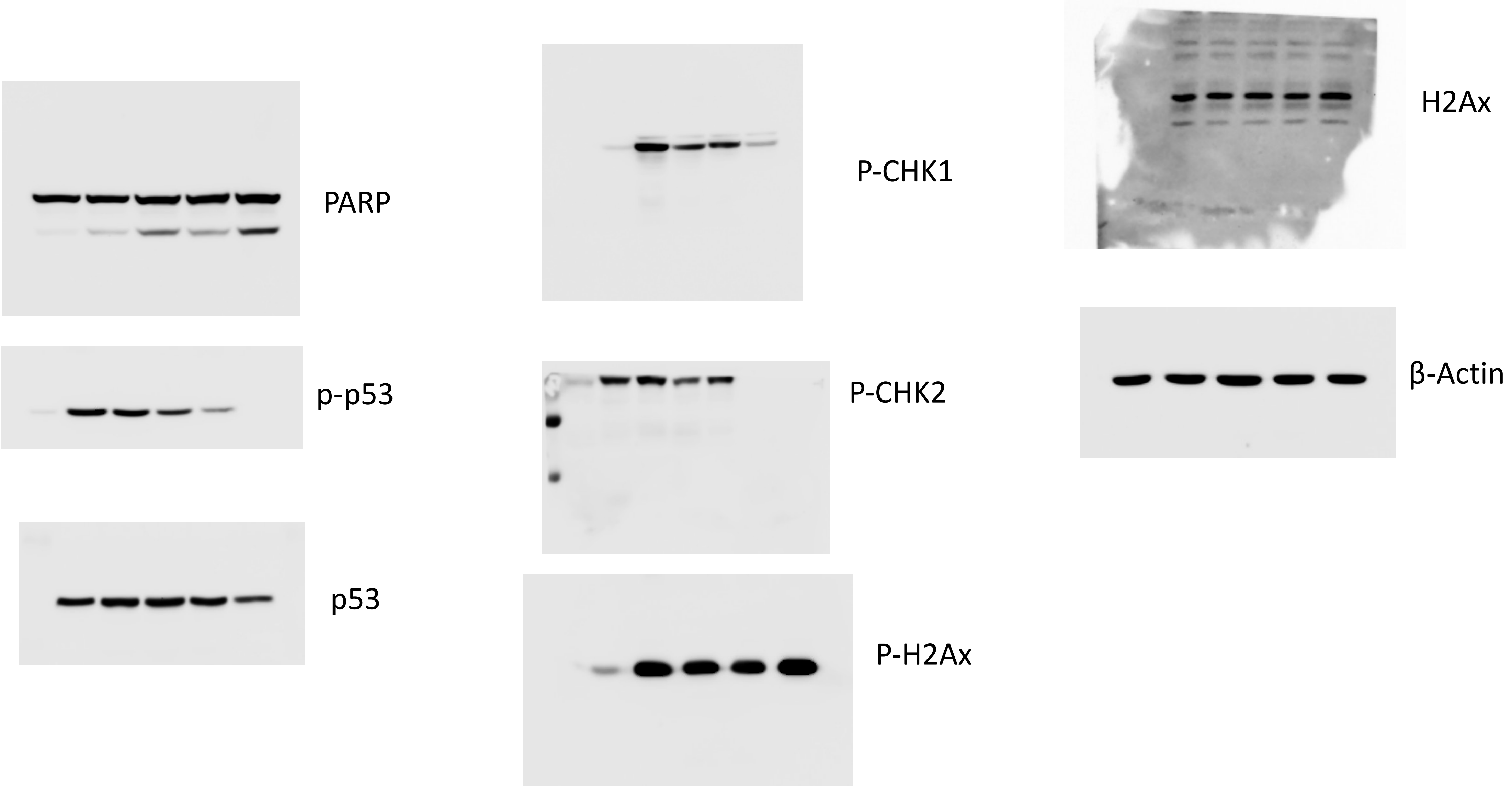

Figure 1B

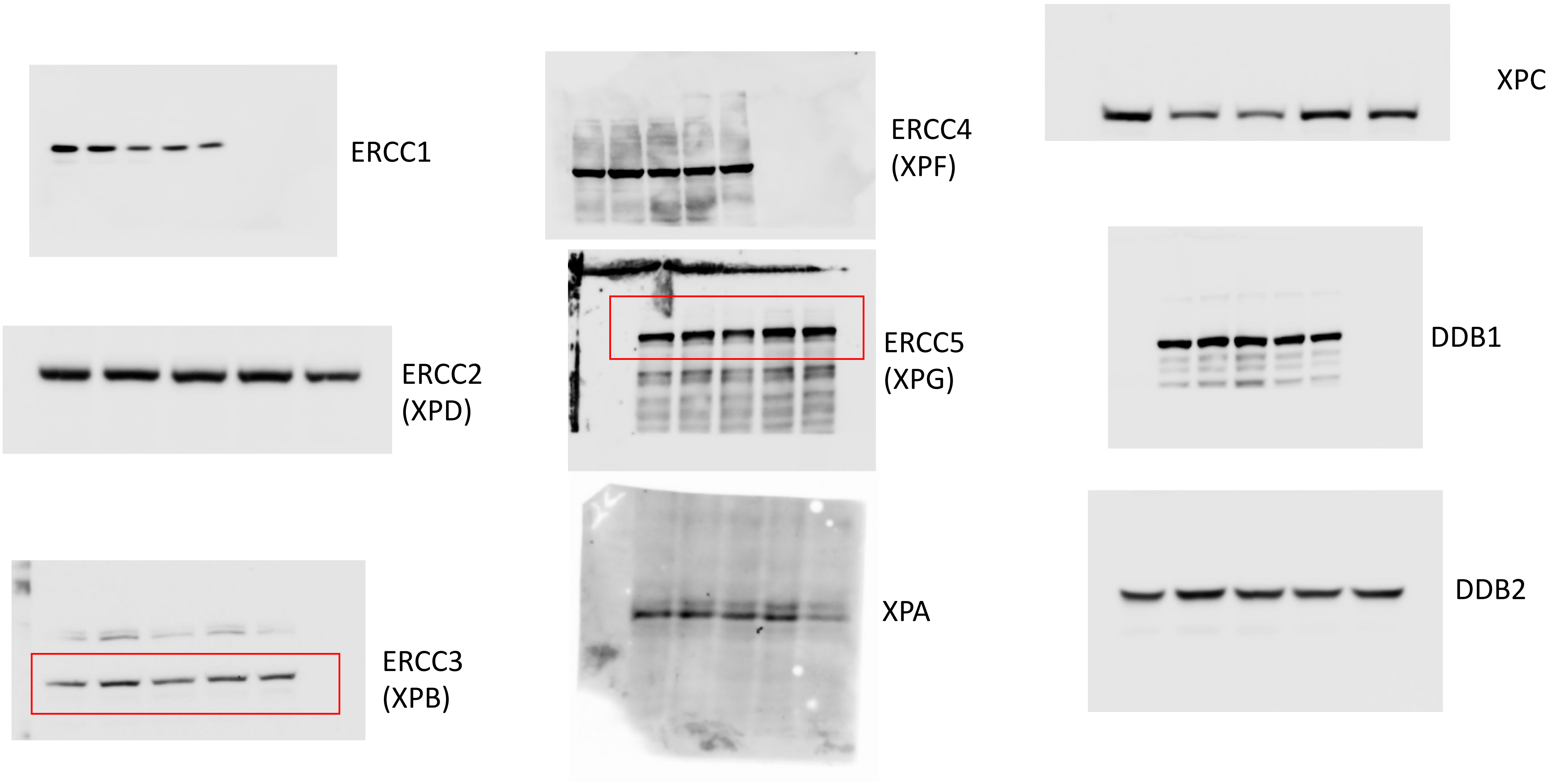
ERCC1
ERCC4 (XPF)
XPC
ERCC2 (XPD)
ERCC5 (XPG)
DDB1
ERCC3 (XPB)
XPA
DDB2

Figure 2

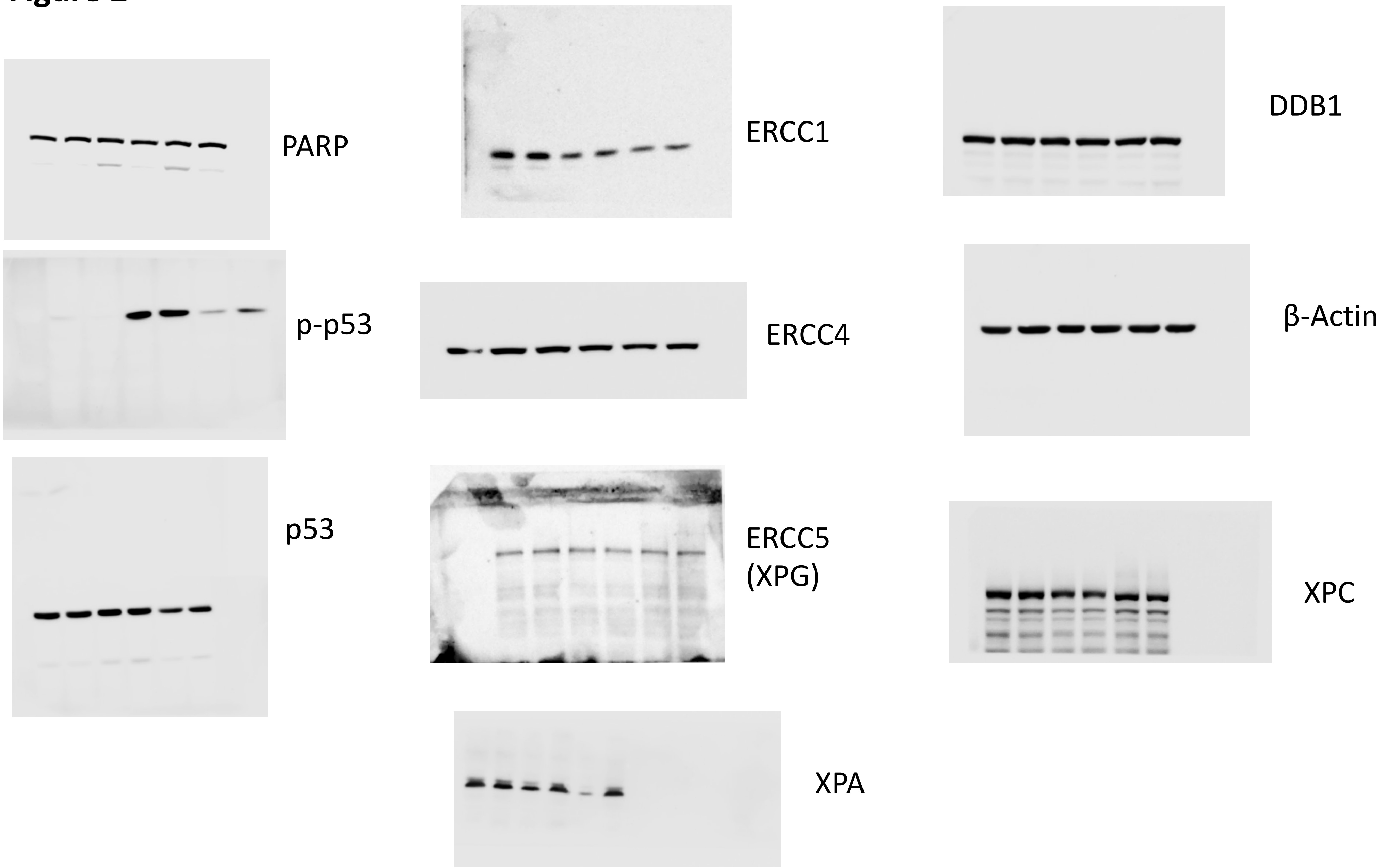

Figure 3A

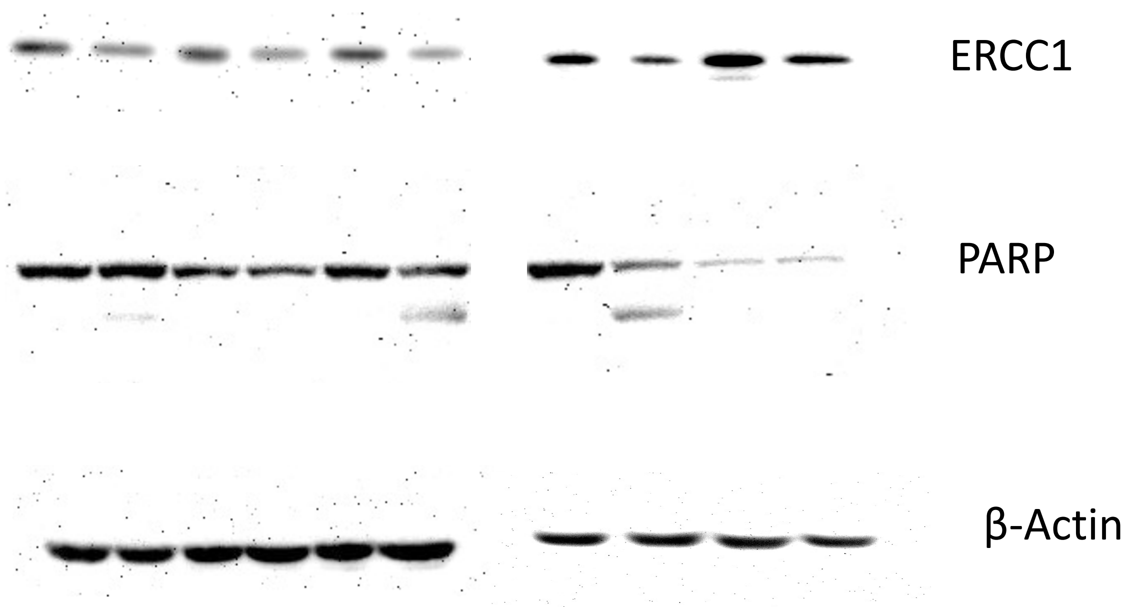

Figure 3B

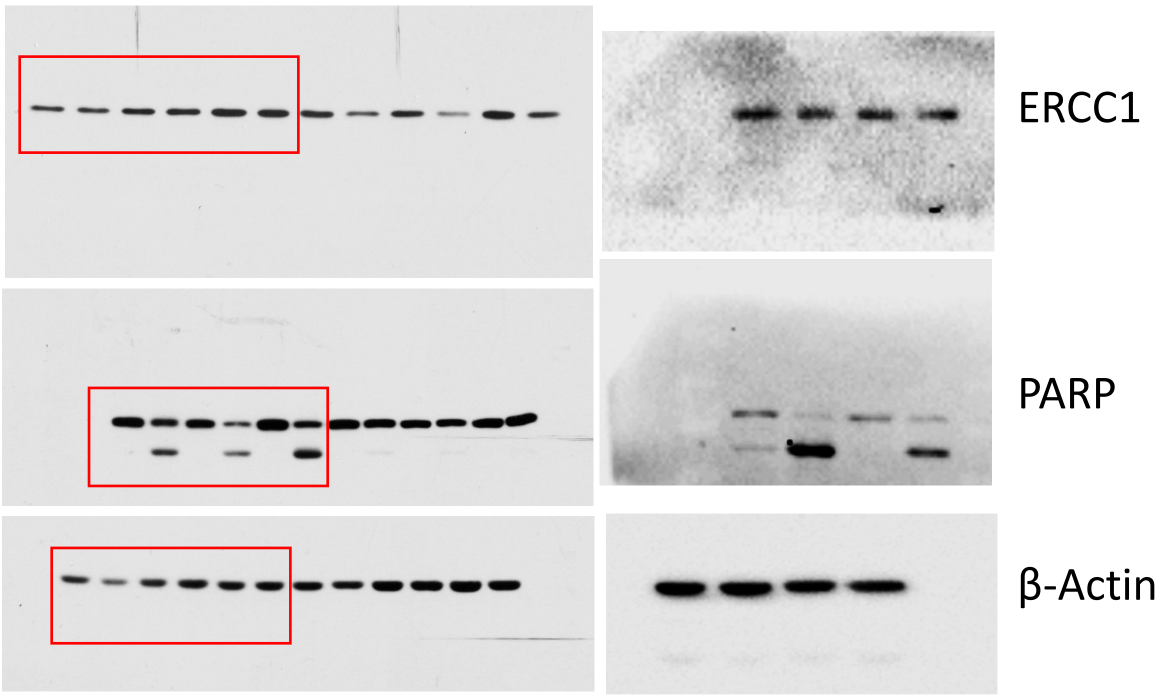

Figure 4A

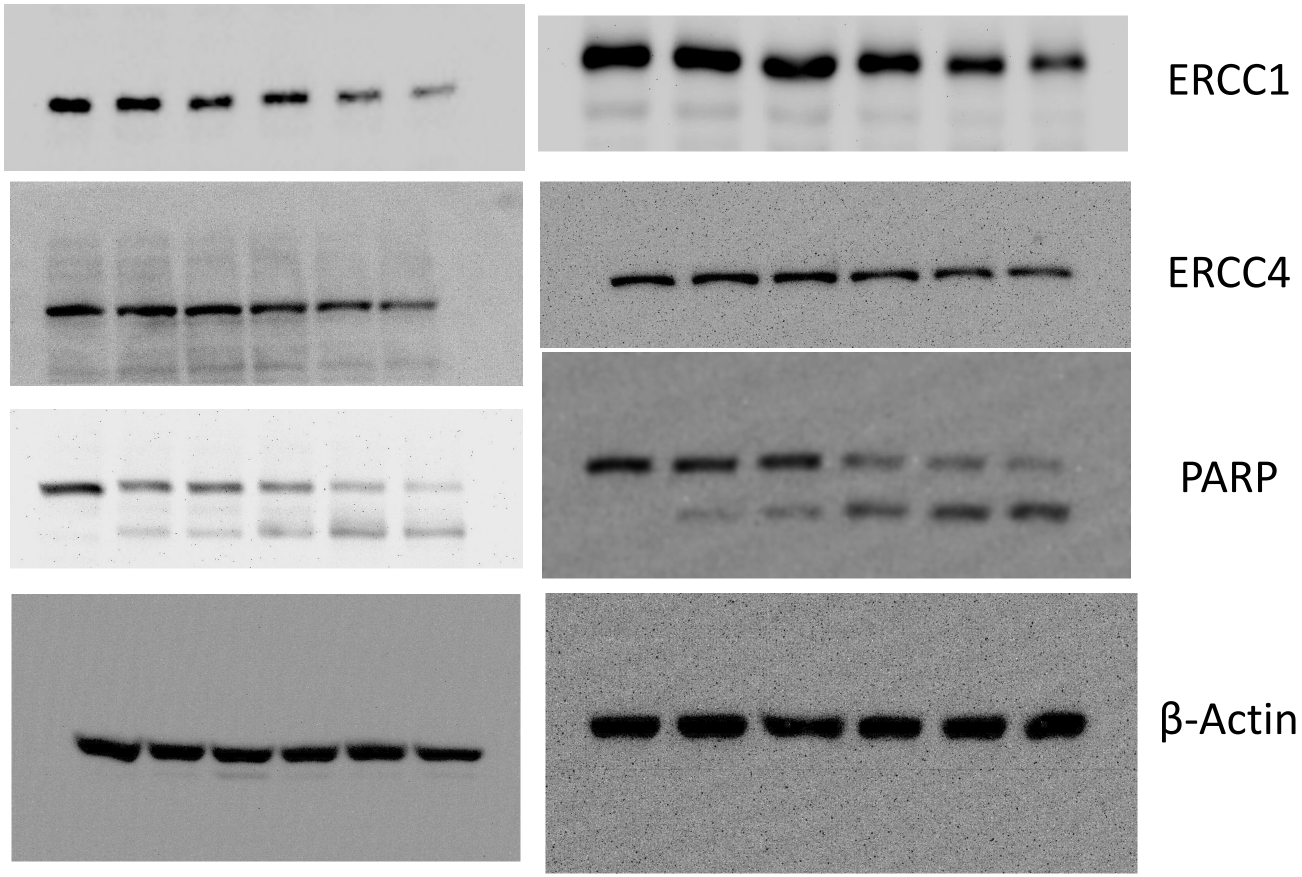

Figure 4B

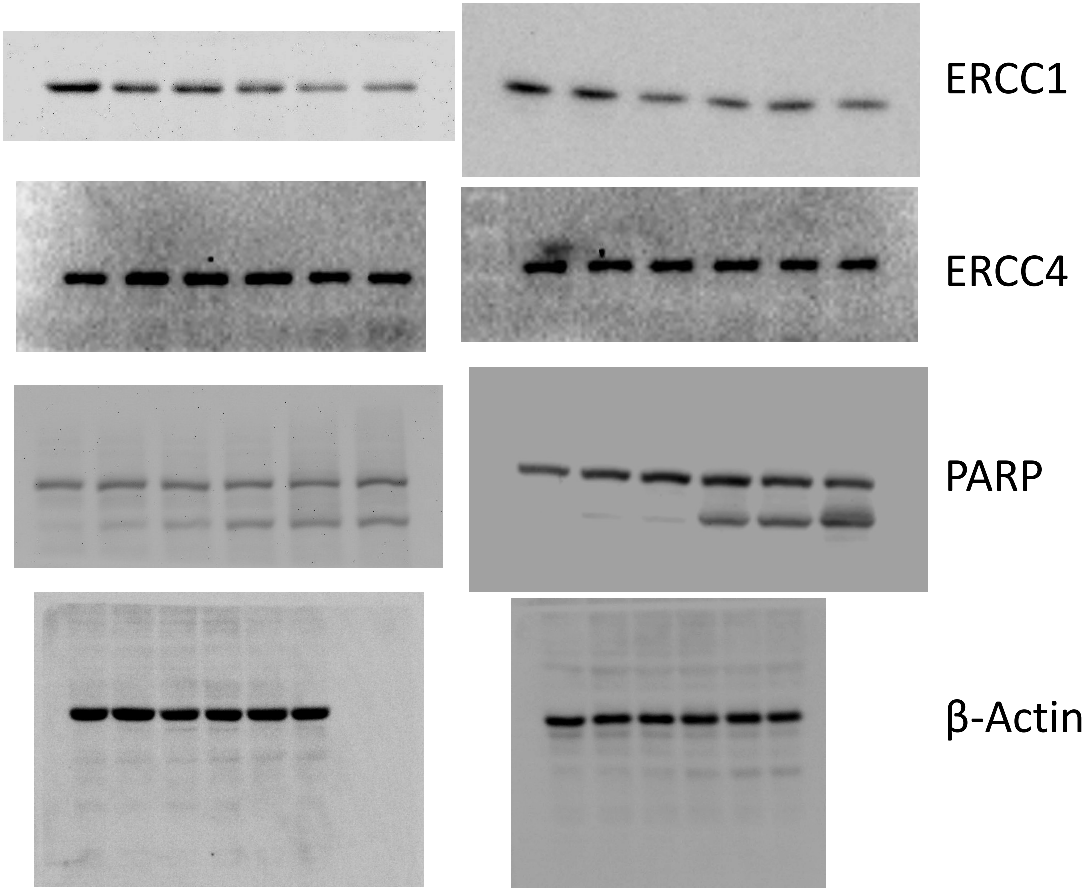

Figure 5B

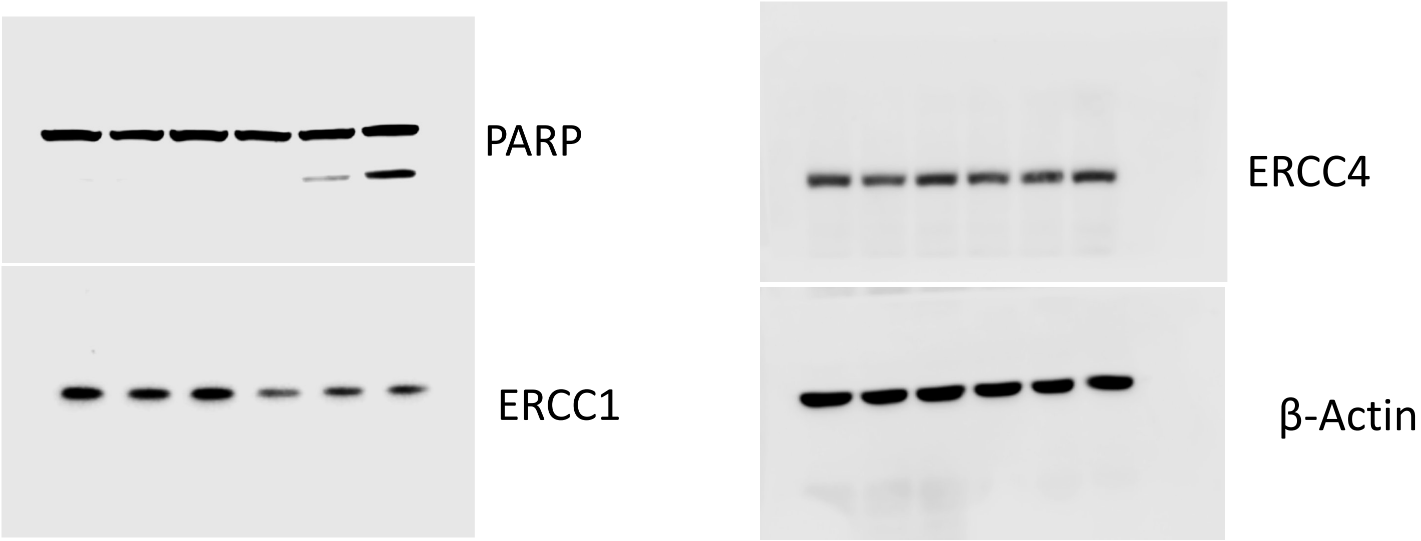

Figure 6A

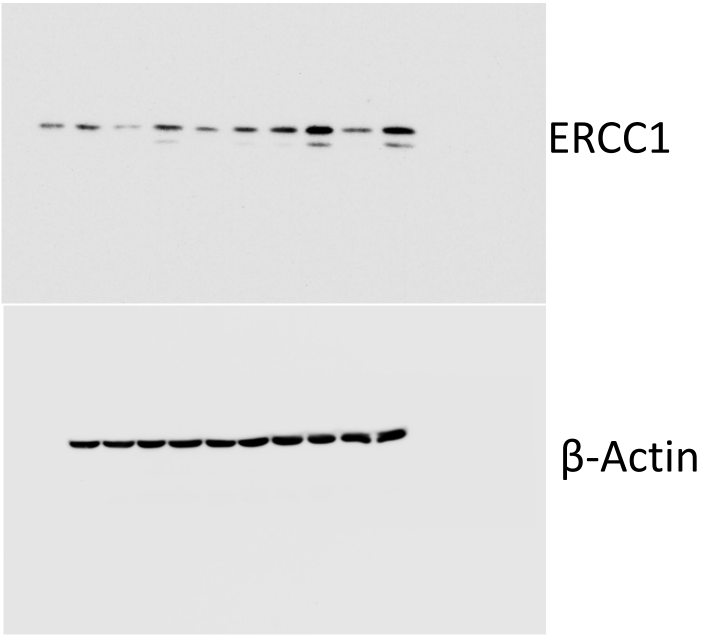

Figure 6B

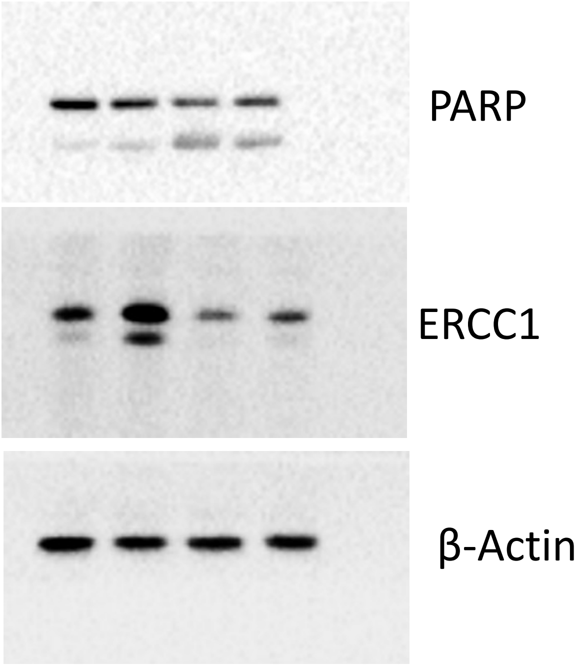

Figure 6C

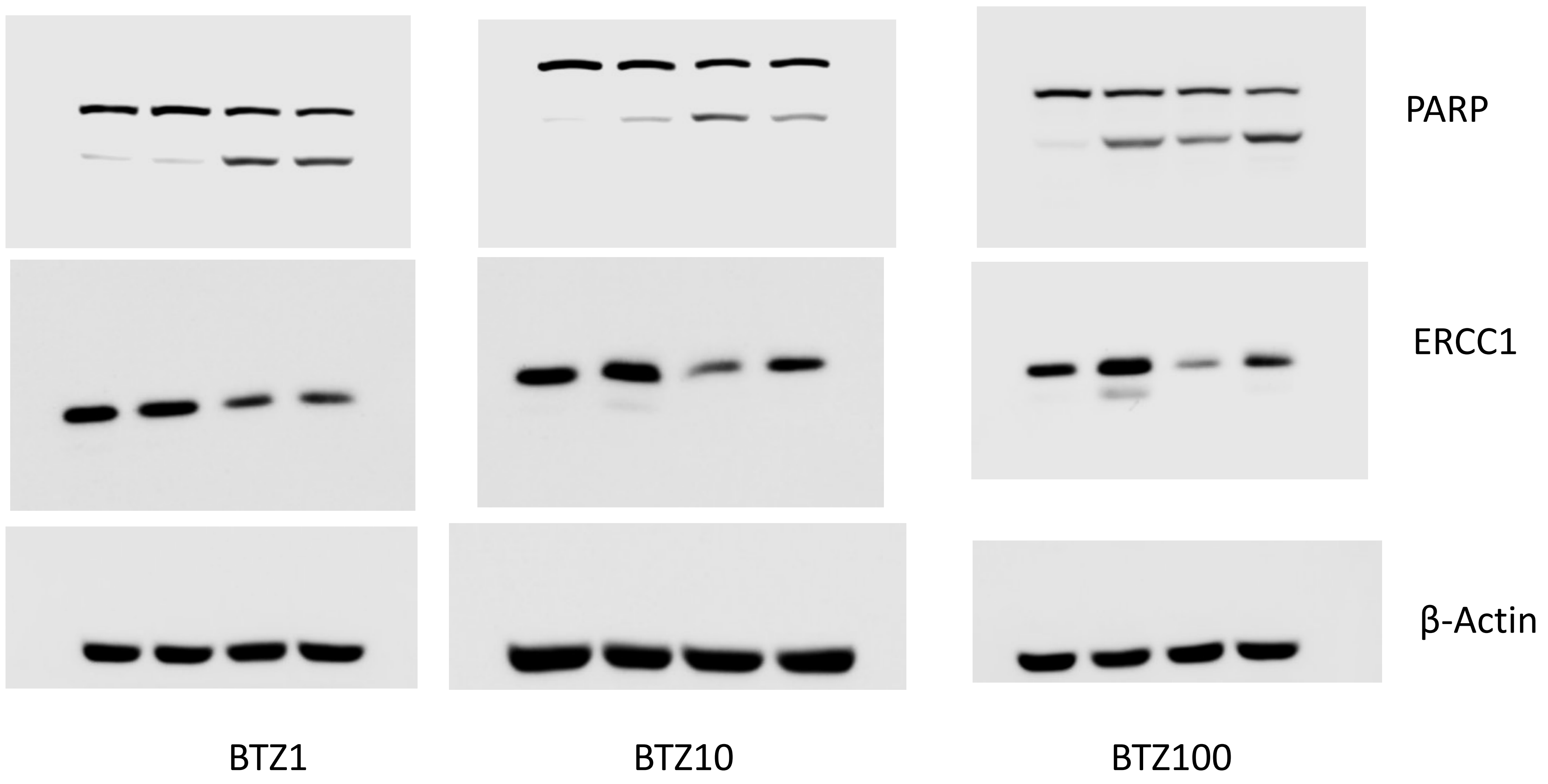

Supplement: Supplementary file 2 — Original Data File [file 41420_2024_1817_MOESM2_ESM.pdf]
